# The economic cost and patient-reported outcomes of chronic Achilles tendon ruptures

**DOI:** 10.1186/s40634-020-00277-z

**Published:** 2020-08-03

**Authors:** Niklas Nilsson, Katarina Nilsson Helander, Eric Hamrin Senorski, Anna Holm, Jón Karlsson, Mikael Svensson, Olof Westin

**Affiliations:** 1 Department of Orthopaedics, Institute of Clinical Sciences at Sahlgrenska Academy, University of Gothenburg, Gothenburg, Sweden; 2 Department of Orthopaedics, Sahlgrenska University Hospital, Mölndal, Sweden; 3 Department of Health and Rehabilitation, Institute of Neuroscience and Physiology, The Sahlgrenska Academy, University of Gothenburg, Gothenburg, Sweden; 4 Health Metrics, The Sahlgrenska Academy, University of Gothenburg, Gothenburg, Sweden

**Keywords:** Achilles tendon rupture, Chronic Achilles tendon rupture, ATRS, Operative

## Abstract

**Purpose:**

While most Achilles tendon ruptures are dramatic and diagnosed quickly, some are missed, with a risk of becoming chronic. A chronic Achilles tendon rupture is defined as a rupture that has been left untreated for more than 4 weeks. By mapping the health economic cost of chronic Achilles tendon ruptures the health-care system might be able to better distribute resources to detect these ruptures at an earlier time.

**Method:**

All patients with a chronic Achilles tendon rupture who were treated surgically at Sahlgrenska University Hospital or Kungsbacka Hospital between 2013 and 2018 were invited to participate in the study. The patients were evaluated postoperatively using the validated Achilles tendon Total Rupture Score (ATRS). The health-care costs were assessed using clinical records. The production-loss costs were extracted from the Swedish Social Insurance Agency. The cost of chronic Achilles tendon ruptures was then compared with the cost of acute ruptures in a previous study by Westin et.al.

**Results:**

Forty patients with a median (range) age of 66 (28–86) were included in the study. The mean total cost (± SD) for the patients with a chronic Achilles tendon rupture was 6494 EUR ± 6508, which is 1276 EUR higher than the mean total cost of acute ruptures. Patients with chronic Achilles tendon ruptures reported a mean (min-max) postoperative ATRS of 73 (14–100).

**Conclusion:**

Missing an Achilles tendon rupture will entail higher health-care costs compared with acute ruptures. Health-care resources can be saved if Achilles tendon ruptures are detected at an early stage.

## Introduction

The Achilles tendon is the strongest tendon in the human body [6]. It is, however, one of the most commonly injured [12]. The incidence of Achilles tendon rupture is approximately 23–47 per 100,000 person-years for men and 8–12 per 100,000 person-years for women [9, 11]. The incidence has increased over the last few decades [7, 9]. The most probable explanation is that more people exercise and participate in sports at an older age. Due to the strength of the Achilles tendon, the rupture is commonly dramatic and is characterized by a loud “snap” and acute pain [10]. There are also ruptures that have a more discrete debut [20]. The atypical presentations of Achilles tendon ruptures are more frequent in the elderly and sedentary population and they are also the ones that are usually missed and become chronic [14].

A chronic Achilles tendon rupture is by the literature defined as a rupture that has been left untreated for more than 4 weeks after the initial injury [8, 17]. The reason why the rupture remains untreated depends on the event, the patient’s experience of the injury and the clinical examination [16]. Of all Achilles tendon ruptures, 10–25% are missed and therefore risk becoming chronic [3, 20]. Patients with chronic Achilles tendon ruptures more frequently complain about pain, recurrent swelling, an affected gait pattern and an inability to climb stairs [13].

A variety of surgical techniques have been described to treat chronic Achilles tendon ruptures. Examples include augmentations, V-Y tendon reconstruction, free flaps, tendon transfers and turndown flaps [1, 13, 21]. In contrast to an acute Achilles tendon rupture, chronic ruptures, are suggested to require a surgical intervention [17]. In most cases, the surgical intervention of a chronic Achilles tendon rupture is more demanding and is associated with greater risks than the intervention in an acute Achilles tendon rupture [19]. Normal end-to-end sutures are not regarded as an acceptable treatment and some kind of reinforcement is commonly recommended [1, 2, 15].

Many studies have reported different surgical methods after chronic Achilles tendon ruptures [13, 18, 21, 24]. However, there are no studies that have evaluated the economic cost of chronic Achilles tendon ruptures. The purpose of this study was to analyze the economic cost of chronic Achilles tendon ruptures. A secondary aim was to present the pre- and postoperative ankle function in patients with chronic Achilles tendon ruptures using the Achilles tendon Total Rupture Score (ATRS).

## Material and methods

### Data collection and study population

The patients in this study were all presented at Sahlgrenska University Hospital and Kungsbacka Hospital between 2013 and 2018. The included patients were found through identifying all patients with Achilles tendon ruptures that were operatively treated at the designated hospitals during the selected time period. The medical history and clinical evaluation was later examined for all identified patients. The criterion for inclusion was any type of unilateral Achilles tendon rupture that had been left untreated for more than 4 weeks and could therefore be classified as chronic. The diagnosis was based on the trauma described, the clinical examination and, in some cases, inspection with ultrasonography. Due to the rarity of chronic Achilles tendon ruptures, no exclusion criteria were used. Fifty-nine patients were identified. Every patient received a letter with information about the study and two Achilles tendon Total Rupture Score questionnaires (ATRS) to evaluate their pre- and postoperative ankle function. Both ATRS questionnaires were filled out retrospectively at the same time by the patient 1 year after surgical repair. Out of the patients identified, 40 patients chose to participate, and got included in the study. All the included patients gave their written consent to take part in the study. Ethical approval was obtained from the regional ethics review board in Sweden (DNR 554–15).

The health-economic cost was calculated as the sum of the surgical costs, other health-care costs related to hospital stays and complications, and the production loss due to sick leave. The health-care costs were extracted from the accounting database at Sahlgrenska University Hospital and the production loss (sick leave) was collected from the Swedish Social Insurance Agency. The health-care costs used in this study are presented in Table 1. The data was later compared with statistics from the economic study performed by Westin et al. [27]. In that study, the economic cost of the operative and nonoperative treatment of acute Achilles tendon ruptures at the same Sahlgrenska University Hospital between 2009 and 2010 were analyzed. The extracted data were collected from the randomized controlled study performed by Olsson et al. [23]. The health-care and production-loss cost of chronic Achilles tendon ruptures in the present study were examined in comparison with the cost of both the operative and nonoperative treatment of acute Achilles tendon ruptures. The costs of physical therapy visits were excluded due to a high number of patients going to privately financed physiotherapists. The function was evaluated using the ATRS.

**Table 1:** The health-economic costs included in the study

Item	Cost per unit (euro)
Accident and Emergency visit	220.99 €
Inpatient night	567.38 €
Surgeon cost/min	5.98 €
Operating room cost/min	17.16 €
Orthopedic out-patient visit	220.99 €
Day-care surgery bed	282.17 €
Ankle brace	203.16 €

### Treatment method

All the patients included in the study were treated operatively. The surgical method used was the surgical technique described by Nilsson-Helander et al. [21] The method is based on the augmentation of the ruptured tendon with a free gastrocnemius aponeurosis flap. Following surgery, the patient’s foot was placed in an equinus position with a below-the-knee plaster cast. This cast was worn for 3 weeks, followed by 3 weeks in a more natural position. After 6 weeks the patients were able to start range-of-motion training in an adjustable ankle brace (DonJoy ROM Walker) for a further 2 weeks. Weight-bearing was successively increased during this period. The total number of days with ankle immobilization was thus 8 weeks.

### Economic costs

The economic costs were classified as either health-care costs or production losses. The direct health-care costs were based on costs extracted from the accounting database at Sahlgrenska University Hospital. The expenses included the cost of administration, hospital wages, surgeon salaries, anesthesia and inpatient nights. Table 1 shows the health-care costs that were considered in the study. The economic costs associated with the production loss depend on the number of sick-leave days and the gross wage of the patient. All the costs were converted from SEK to EUR using the 2013 exchange rate (1 EUR = 8.86 SEK = 1.33 USD).

### Patient-reported outcomes

This study uses the validated Achilles tendon Total Rupture Score (ATRS) [22] to evaluate functional outcomes among patients with a chronic Achilles tendon rupture.

### Statistical analysis

The economic costs were expressed in euros and analyzed as continuous variables summarized in the arithmetic mean and standard deviation and 95% confidence intervals. Parametric statistical tests for significant differences in economic costs between chronic and acute ruptures were based on the assumption of normally distributed data. Sensitivity checks of statistical significance tests were performed using non-parametric bootstrapping. *P*-values below 0.05 were considered statistically significant.

## Results

### Demographics

A total of 40 consecutive patients decided to participate in the study (29 males and 11 females). The median age (range) was 66 (28–86). Table 2 presents a summary of the demographic and clinical variables. The demographic and clinical variables of patients who did not choose to participate (non-response) are also presented. There were no evident demographic differences between the two groups.

**Table 2:** Summary of statistics relating to demographic and clinical variables of interest for included patients and non-response patients. Categorical variables are presented as n (%) and continuous variables as the mean (± SD)

Item	Total response (***n*** = 40)	Non-response (***n*** = 19)
**Patient gender**		
Male	29 (72.5%)	17 (89.5%)
Female	11 (27.5%)	2 (10.5%)
**Age**	62.7 ± 13.8	50.0 ± 17.0
**Hospital admission**		
Yes	33 (82.5%)	18 (94.7%)
No	7 (17.5%)	1 (5.3%)
**Physician visits**	4.7 ± 1.6	4.7 ± 3.0
**Operative time (minutes)**	85.6 ± 20.8	92.7 ± 22.0

### Economic cost

Table 3 presents the mean (CI 95%; lower-upper) economic cost per patient for chronic Achilles tendon ruptures in terms of health-care, production losses and total costs. The mean (± SD) health-care cost of chronic Achilles tendon ruptures in the study was 3821 EUR ± 752. When combined with the production-loss cost of chronic Achilles tendon ruptures, this equals a mean (± SD) total cost of 6494 EUR ± 6508. The production-loss cost presented here includes both working patients and patients that have retired from work. When working patients (*n* = 22) were analyzed exclusively, the mean (± SD) production-loss cost was 6831 EUR ± 4861 instead of 2673 EUR ± 6625. The total cost of chronic Achilles tendon ruptures is therefore considerably higher when it affects working patients.

**Table 3:** Comparison of the mean (CI 95%; lower–upper) economic cost (EUR) per patient between chronic Achilles tendon ruptures and the operative and nonoperative treatment of acute Achilles tendon ruptures. **Significant difference compared to chronic Achilles tendon ruptures is presented by bold font**

Variable	Health-care costs	Production-loss costs	Total costs
Chronic rupture	3821 (3580 – 4061)	2673 (555–4792)	6494 (4413 – 8576)
Nonoperative treatment of acute rupture	**742 (696–787)**	3730 (2230 – 5230)	**4472 (2972 – 5971)**
Operative treatment of acute rupture	**3146 (2986 – 3306)**	2853 (1728 – 3978)	5999 (4862 – 7135)

**Fig. 1:**
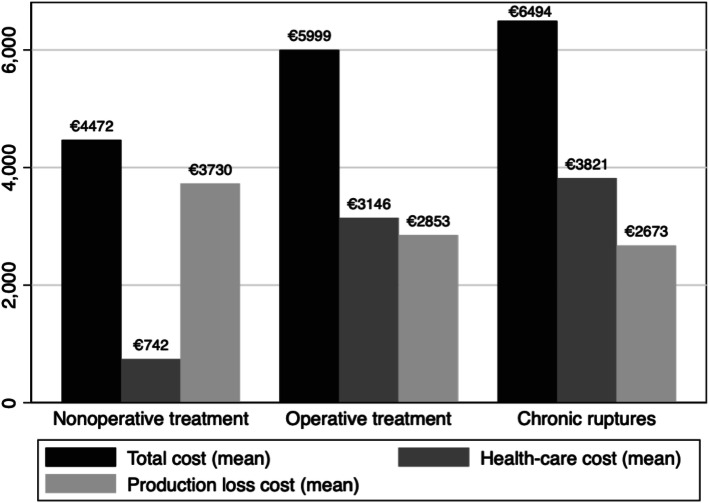
Diagram depicting the differences in health-care, production-loss and total costs (EUR) of chronic and the surgical and non-surgical treatment of acute Achilles tendon ruptures

Table 3 also shows a comparison between the cost per patient of chronic Achilles tendon ruptures and acute Achilles tendon ruptures. All the results are adjusted for gender and age. The results demonstrate a higher health-care cost for chronic Achilles tendon ruptures than both the operative and nonoperative treatment of acute Achilles tendon ruptures. The mean difference in health-care costs between chronic ruptures and the nonoperative treatment of an acute rupture was 3079 EUR and between chronic ruptures and the operative repair of an acute rupture 675 EUR. All differences regarding health-care costs were statistically significant (*P* < 0.01). Table 3 also presents production-loss and total costs per patient for each group. There were no significant differences in production-loss costs between the three groups (Fig. 1).

When comparing the total cost, regardless of treatment, of chronic Achilles tendon ruptures and acute Achilles tendon ruptures, a non-significant mean cost difference of 1276 EUR (*P*-value = 0.11) is seen.

### Patient-reported outcome

Patients with a chronic Achilles tendon rupture reported an improvement in the ATRS from initial injury to 1 year after treatment. The mean (± SD) preoperative ATRS was 16.2 ± 13.0 and the mean (± SD) postoperative ATRS was 73.2 ± 22.7, (*P* < 0.001). The scores are presented in Table 4.

**Table 4:** Mean retrospective preoperative and postoperative ATRS among patients with chronic Achilles tendon ruptures. The mean difference between the two variables is also presented

ATRS	Mean	SD	95% CI (lower-upper)	Range (min-max)
Preoperative	16.2	13.0	12.5 20.9	0.0–62.0
Postoperative	73.3	22.8	64.0 79.5	14.0–100.0
Difference	57.1	22.5	49.9 64.2	10.0–91.0

## Discussion

The most important finding of this study is that chronic Achilles tendon ruptures are more expensive than acute Achilles tendon ruptures. Another important finding was that patients with chronic Achilles tendon ruptures reported improved ankle function 1 year after operative repair and rehabilitation. This indicates that the treatment of chronic Achilles tendon ruptures is effective, even though it requires more resources than the treatment of acute Achilles tendon ruptures. The direct health-care costs were higher for chronic Achilles tendon ruptures, regardless of whether the acute Achilles tendon ruptures were treated operatively or nonoperatively. It can be assumed that the reason for the higher costs is the more complicated surgical technique required when treating chronic Achilles tendon ruptures. The more difficult procedure results in longer operating times and higher costs.

When analyzing the health-economic cost of an injury, it is important to consider the patient-reported outcome. The mean postoperative ATRS reported by patients with chronic Achilles tendon ruptures was 73 points of 100. The mean preoperative ATRS was 16. This indicates that the surgical repair of chronic Achilles tendon ruptures will result in significantly improved ankle function. In comparison, patients surgically treated for an acute Achilles tendon rupture at the same hospitals reported a slightly higher ATRS of 82 points 12 months after the initial injury [23]. The non-surgically treated patients scored 80 in the same trial. This means that, in addition to a higher economic cost, chronic Achilles tendon ruptures may be associated with more patient-reported limitations in ankle function than acute Achilles tendon ruptures. There is no official MCID (Minimal Clinically Important Difference) for ATRS. However, earlier studies have defined the MCID as 8–10 points [5, 26]. Both the preoperative and the postoperative ATRS were filled out retrospectively. This entails an immense recall bias. This is a notable drawback of the study.

Prior to this study, there have only been a few health-economic studies that have evaluated the economic cost of Achilles tendon ruptures. Truntzer et al. [25] and Westin et al. [27] have evaluated whether operative or nonoperative treatment is economically favorable when it comes to Achilles tendon ruptures. Moreover, Carmont et al. [4] compared the economic impact of the open and percutaneous repair of Achilles tendon ruptures.

The study performed by Truntzer et al. [25] showed that the cost of a nonoperative approach to acute Achilles tendon ruptures was significantly lower than that of an operative approach. However, the study was limited to direct costs and did not consider the economic impact of performance loss and costs related to quality of life. As a result, the costs that were considered were only analyzed from the perspective of health-care and not of the patient. Westin et al. [27] therefore conducted a health-economic study where production-loss costs (indirect costs) and the cost per gained QALY were examined. The study reported that operative treatments are more expensive, but that they could be regarded as equally cost effective if there is a willingness to pay 50,000 EUR/QALY. The cost of re-ruptures was not included. Carmont et al. [4] reported that the percutaneous repair of the Achilles tendon is a cost-effective alternative to open repair of the tendon. Chronic Achilles tendon ruptures are still thought to require an open repair, as described in the study by Nilsson-Helander et al. [21]. They evaluated the functional- and patient-reported outcome of a surgical method applied to both chronic Achilles tendon ruptures and re-ruptures. They did not, however, consider economic costs and did not analyze chronic Achilles tendon ruptures exclusively.

The limitation of this study is that the age of patients with chronic Achilles tendon ruptures is considerably higher than that of patients with acute Achilles tendon ruptures. Consequently, more patients have retired from work and therefore lack information about loss of income due to sick leave and fewer working hours. This results in lower production-loss costs and thereby a lower total cost for these ruptures. When exclusively analyzing the production-loss cost for working patients, the cost was considerably higher. Older age may potentially also have affected the patient-reported outcome and ATRS. This is the reason why no direct comparison regarding ATRS, between chronic Achilles tendon ruptures and acute Achilles tendon ruptures, was made. To determine the total cost difference between acute and chronic Achilles tendon ruptures and their patient-reported outcome, a larger cohort is required. The persistent challenge is that chronic Achilles tendon ruptures are relatively uncommon and thereby difficult to recruit for studies on a larger scale. Another limitation is that physiotherapy costs had to be excluded. Patients with chronic Achilles tendon ruptures often need an extensive time to rehabilitate. However, due to the age difference between the groups, the patients with chronic Achilles tendon ruptures may have lower functional requirements than the younger group of patients with acute Achilles tendon rupture. Therefore, the exclusion of physiotherapy visits, might affect the result in both ways. For a more precise analysis, an inclusion of physiotherapy costs would be eligible.

The health-economic costs analyzed in this study are based on the Swedish health-care system and may not be applicable to other countries with different health-care structures. Due to the limited number of females included in the study, no gender comparison was performed. The strength of the study is, however, that it analyzes chronic Achilles tendon ruptures exclusively.

## Conclusion

The treatment of patients with a chronic Achilles tendon rupture is more expensive than the treatment of acute Achilles tendon ruptures. The main reason is the significantly higher costs of operative intervention. Moreover, the operative repair of chronic Achilles tendon ruptures improved the ankle function among patients. Patients with chronic Achilles tendon ruptures might, however, still have an inferior patient-reported outcome compared to patients with acute Achilles tendon ruptures. This indicates that patients with chronic Achilles tendon ruptures have persistent limitations after surgery and that more resources are required to detect these ruptures at an earlier stage.

## Data Availability

Data will not be shared. All data used is presented in the manuscript.
